# Supplementary material for: Elucidating the causal role of age of menarche, adiposity, lipid fractions, and blood pressure upon cardiovascular disease: a multivariable Mendelian randomization study
Source: J Hum Hypertens. 2025 Aug 19;39(10):723–34. doi: 10.1038/s41371-025-01048-y (PMC12500469; doi:10.1038/s41371-025-01048-y)
Supplement: Supplementary file 1 — journal of human hypertension_Supplementary Material_jyongho_revised.2024.10 [file 41371_2025_1048_MOESM1_ESM.docx]

Supplementary Material

Additional materials including code for replicating analyses are available at:

<https://github.com/WSpiller/CVDMVMR>

S1: Descriptive statistics for UKB GWAS sample

| **Risk Factor** | **Mean (sd)** | **N (%)** |
| --- | --- | --- |
| Age of menarche  Age in years | 12.84 (1.58) |  |
| Alcohol  None  Special occasions only  One to three times a month  Once or twice a week  Three or four times a week  Daily or almost daily |  | 7,198 (7.56)  10,535 (11.06)  11,106 (11.66)  24,522 (25.74)  22,418 (23.53)  19,491 (20.46) |
| BMI  $kg/m^{2}$ | 27.23 (4.77) |  |
| Blood pressure  DBP $mg/dL$  SBP $mg/dL$ | 81.32 (10.54)  137.92 (19.38) |  |
| Creatinine in urine  $mg/dL$ | 100.32 (65.53) |  |
| Incident Ischemic stroke  Positive |  | 9,527 (10.00) |
| Lipid fractions  HDL $mg/dL$  LDL $mg/dL$  Triglycerides $mg/dL$ | 1.44 (0.38)  3.54 (0.86)  1.75 (1.01) |  |
| Sex  Female |  | 52397 (55) |
| Smoking  Never  Former  Current |  | 57,037 (59.87)  27,845 (29.23)  10,388 (10.90) |
| Sleep duration  Average hours per day | 7.13 (1.11) |  |
| Type-II diabetes  Positive |  | 5,097 (5.35) |
| Vigorous activity p/week  Days | 1.83 (1.94) |  |

S2: Heterogeneity statistics for univariable IVW analyses. ID numbers represent identification numbers with reference to GWAS summary data obtained using the OpenGWAS platform.

| **Exposure** | **Outcome** | **Number of SNPs** | **Q** | **p-value** |
| --- | --- | --- | --- | --- |
| Age when periods started (menarche) \|\| id:ukb-a-315 | Coronary heart disease \|\| id:ieu-a-7 | 119 | 205.7519 | 1.03E-06 |
| Alcohol consumption \|\| id:ieu-a-1283 | Coronary heart disease \|\| id:ieu-a-7 | 4 | 14.90229 | 0.001902 |
| Body mass index (BMI) \|\| id:ukb-b-19953 | Coronary heart disease \|\| id:ieu-a-7 | 425 | 665.9838 | 4.99E-13 |
| Diastolic blood pressure automated reading \|\| id:ukb-a-359 | Coronary heart disease \|\| id:ieu-a-7 | 172 | 570.1769 | 2.00E-44 |
| Systolic blood pressure automated reading \|\| id:ukb-a-360 | Coronary heart disease \|\| id:ieu-a-7 | 156 | 682.4143 | 3.04E-67 |
| Creatinine (enzymatic) in urine \|\| id:ukb-a-333 | Coronary heart disease \|\| id:ieu-a-7 | 20 | 47.95126 | 0.000261 |
| HDL cholesterol \|\| id:ieu-b-109 | Coronary heart disease \|\| id:ieu-a-7 | 318 | 855.9904 | 4.03E-51 |
| LDL cholesterol \|\| id:ieu-b-110 | Coronary heart disease \|\| id:ieu-a-7 | 146 | 789.7624 | 2.41E-89 |
| triglycerides \|\| id:ieu-b-111 | Coronary heart disease \|\| id:ieu-a-7 | 275 | 742.7779 | 6.89E-45 |
| Past tobacco smoking \|\| id:ukb-b-2134 | Coronary heart disease \|\| id:ieu-a-7 | 91 | 156.8127 | 1.66E-05 |
| Sleep duration \|\| id:ukb-b-4424 | Coronary heart disease \|\| id:ieu-a-7 | 65 | 100.9629 | 0.002206 |
| Type 2 diabetes \|\| id:ieu-a-26 | Coronary heart disease \|\| id:ieu-a-7 | 10 | 10.8634 | 0.285188 |
| Number of days/week of vigorous physical activity 10+ minutes \|\| id:ukb-a-511 | Coronary heart disease \|\| id:ieu-a-7 | 4 | 1.650823 | 0.647922 |
| Age when periods started (menarche) \|\| id:ukb-a-315 | Ischemic stroke (small-vessel) \|\| id:ebi-a-GCST006909 | 120 | 157.2831 | 0.010766 |
| Alcohol consumption \|\| id:ieu-a-1283 | Ischemic stroke (small-vessel) \|\| id:ebi-a-GCST006909 | 4 | 0.80211 | 0.848962 |
| Body mass index (BMI) \|\| id:ukb-b-19953 | Ischemic stroke (small-vessel) \|\| id:ebi-a-GCST006909 | 437 | 528.4932 | 0.001554 |
| Diastolic blood pressure automated reading \|\| id:ukb-a-359 | Ischemic stroke (small-vessel) \|\| id:ebi-a-GCST006909 | 174 | 258.47 | 2.72E-05 |
| Systolic blood pressure automated reading \|\| id:ukb-a-360 | Ischemic stroke (small-vessel) \|\| id:ebi-a-GCST006909 | 161 | 270.7207 | 1.05E-07 |
| Creatinine (enzymatic) in urine \|\| id:ukb-a-333 | Ischemic stroke (small-vessel) \|\| id:ebi-a-GCST006909 | 21 | 35.58815 | 0.017186 |
| HDL cholesterol \|\| id:ieu-b-109 | Ischemic stroke (small-vessel) \|\| id:ebi-a-GCST006909 | 321 | 406.2779 | 0.000761 |
| LDL cholesterol \|\| id:ieu-b-110 | Ischemic stroke (small-vessel) \|\| id:ebi-a-GCST006909 | 149 | 202.9038 | 0.001851 |
| triglycerides \|\| id:ieu-b-111 | Ischemic stroke (small-vessel) \|\| id:ebi-a-GCST006909 | 277 | 347.58 | 0.002221 |
| Past tobacco smoking \|\| id:ukb-b-2134 | Ischemic stroke (small-vessel) \|\| id:ebi-a-GCST006909 | 94 | 101.1119 | 0.265366 |
| Sleep duration \|\| id:ukb-b-4424 | Ischemic stroke (small-vessel) \|\| id:ebi-a-GCST006909 | 66 | 75.70336 | 0.171204 |
| Type 2 diabetes \|\| id:ieu-a-26 | Ischemic stroke (small-vessel) \|\| id:ebi-a-GCST006909 | 10 | 8.751057 | 0.460564 |
| Number of days/week of vigorous physical activity 10+ minutes \|\| id:ukb-a-511 | Ischemic stroke (small-vessel) \|\| id:ebi-a-GCST006909 | 4 | 1.830675 | 0.608283 |
| Age when periods started (menarche) \|\| id:ukb-a-315 | Ischemic stroke (large artery atherosclerosis) \|\| id:ebi-a-GCST006907 | 120 | 153.1064 | 0.019161 |
| Alcohol consumption \|\| id:ieu-a-1283 | Ischemic stroke (large artery atherosclerosis) \|\| id:ebi-a-GCST006907 | 4 | 4.686874 | 0.196215 |
| Body mass index (BMI) \|\| id:ukb-b-19953 | Ischemic stroke (large artery atherosclerosis) \|\| id:ebi-a-GCST006907 | 438 | 550.1554 | 0.000184 |
| Diastolic blood pressure automated reading \|\| id:ukb-a-359 | Ischemic stroke (large artery atherosclerosis) \|\| id:ebi-a-GCST006907 | 174 | 248.9487 | 0.000137 |
| Systolic blood pressure automated reading \|\| id:ukb-a-360 | Ischemic stroke (large artery atherosclerosis) \|\| id:ebi-a-GCST006907 | 161 | 286.2122 | 3.44E-09 |
| Creatinine (enzymatic) in urine \|\| id:ukb-a-333 | Ischemic stroke (large artery atherosclerosis) \|\| id:ebi-a-GCST006907 | 21 | 25.06686 | 0.198885 |
| HDL cholesterol \|\| id:ieu-b-109 | Ischemic stroke (large artery atherosclerosis) \|\| id:ebi-a-GCST006907 | 321 | 388.7255 | 0.005095 |
| LDL cholesterol \|\| id:ieu-b-110 | Ischemic stroke (large artery atherosclerosis) \|\| id:ebi-a-GCST006907 | 149 | 227.5594 | 2.87E-05 |
| triglycerides \|\| id:ieu-b-111 | Ischemic stroke (large artery atherosclerosis) \|\| id:ebi-a-GCST006907 | 278 | 309.0131 | 0.090278 |
| Past tobacco smoking \|\| id:ukb-b-2134 | Ischemic stroke (large artery atherosclerosis) \|\| id:ebi-a-GCST006907 | 94 | 110.4435 | 0.104741 |
| Sleep duration \|\| id:ukb-b-4424 | Ischemic stroke (large artery atherosclerosis) \|\| id:ebi-a-GCST006907 | 66 | 116.5276 | 9.16E-05 |
| Type 2 diabetes \|\| id:ieu-a-26 | Ischemic stroke (large artery atherosclerosis) \|\| id:ebi-a-GCST006907 | 10 | 15.23973 | 0.084558 |
| Number of days/week of vigorous physical activity 10+ minutes \|\| id:ukb-a-511 | Ischemic stroke (large artery atherosclerosis) \|\| id:ebi-a-GCST006907 | 4 | 2.441381 | 0.485978 |
| Age when periods started (menarche) \|\| id:ukb-a-315 | Ischemic stroke (cardioembolic) \|\| id:ebi-a-GCST006910 | 120 | 142.0355 | 0.073669 |
| Alcohol consumption \|\| id:ieu-a-1283 | Ischemic stroke (cardioembolic) \|\| id:ebi-a-GCST006910 | 4 | 1.946132 | 0.583662 |
| Body mass index (BMI) \|\| id:ukb-b-19953 | Ischemic stroke (cardioembolic) \|\| id:ebi-a-GCST006910 | 436 | 488.8882 | 0.037625 |
| Diastolic blood pressure automated reading \|\| id:ukb-a-359 | Ischemic stroke (cardioembolic) \|\| id:ebi-a-GCST006910 | 174 | 197.7794 | 0.095297 |
| Systolic blood pressure automated reading \|\| id:ukb-a-360 | Ischemic stroke (cardioembolic) \|\| id:ebi-a-GCST006910 | 161 | 262.8082 | 5.47E-07 |
| Creatinine (enzymatic) in urine \|\| id:ukb-a-333 | Ischemic stroke (cardioembolic) \|\| id:ebi-a-GCST006910 | 21 | 19.81773 | 0.469382 |
| HDL cholesterol \|\| id:ieu-b-109 | Ischemic stroke (cardioembolic) \|\| id:ebi-a-GCST006910 | 319 | 313.4936 | 0.56083 |
| LDL cholesterol \|\| id:ieu-b-110 | Ischemic stroke (cardioembolic) \|\| id:ebi-a-GCST006910 | 147 | 207.0288 | 0.000673 |
| triglycerides \|\| id:ieu-b-111 | Ischemic stroke (cardioembolic) \|\| id:ebi-a-GCST006910 | 277 | 269.425 | 0.600123 |
| Past tobacco smoking \|\| id:ukb-b-2134 | Ischemic stroke (cardioembolic) \|\| id:ebi-a-GCST006910 | 93 | 110.0165 | 0.097034 |
| Sleep duration \|\| id:ukb-b-4424 | Ischemic stroke (cardioembolic) \|\| id:ebi-a-GCST006910 | 66 | 79.40905 | 0.107791 |
| Type 2 diabetes \|\| id:ieu-a-26 | Ischemic stroke (cardioembolic) \|\| id:ebi-a-GCST006910 | 10 | 17.20666 | 0.045576 |
| Number of days/week of vigorous physical activity 10+ minutes \|\| id:ukb-a-511 | Ischemic stroke (cardioembolic) \|\| id:ebi-a-GCST006910 | 4 | 3.221595 | 0.358705 |

S3: Univariable MR analyses for each exposure, including MR-Egger, weighted median, and weighted modal sensitivity analyses. ID numbers represent identification numbers with reference to GWAS summary data obtained using the OpenGWAS platform.

| Exposure | Outcome | Method | Odds ratio | p-value |
| --- | --- | --- | --- | --- |
| Age when periods started (menarche) \|\| id:ukb-a-315 | Coronary heart disease \|\| id:ieu-a-7 | Inverse variance weighted | 0.802088 | 0.000273 |
| Age when periods started (menarche) \|\| id:ukb-a-315 | Coronary heart disease \|\| id:ieu-a-7 | MR Egger | 0.872338 | 0.445059 |
| Age when periods started (menarche) \|\| id:ukb-a-315 | Coronary heart disease \|\| id:ieu-a-7 | Weighted median | 0.82952 | 0.019392 |
| Age when periods started (menarche) \|\| id:ukb-a-315 | Coronary heart disease \|\| id:ieu-a-7 | Weighted mode | 0.84702 | 0.134284 |
| Alcohol consumption \|\| id:ieu-a-1283 | Coronary heart disease \|\| id:ieu-a-7 | Inverse variance weighted | 1.349969 | 0.543991 |
| Alcohol consumption \|\| id:ieu-a-1283 | Coronary heart disease \|\| id:ieu-a-7 | MR Egger | 24.03042 | 0.426609 |
| Alcohol consumption \|\| id:ieu-a-1283 | Coronary heart disease \|\| id:ieu-a-7 | Weighted median | 1.261845 | 0.367162 |
| Alcohol consumption \|\| id:ieu-a-1283 | Coronary heart disease \|\| id:ieu-a-7 | Weighted mode | 1.251305 | 0.427623 |
| Body mass index (BMI) \|\| id:ukb-b-19953 | Coronary heart disease \|\| id:ieu-a-7 | Inverse variance weighted | 1.514478 | 2.17E-29 |
| Body mass index (BMI) \|\| id:ukb-b-19953 | Coronary heart disease \|\| id:ieu-a-7 | MR Egger | 1.683169 | 3.77E-07 |
| Body mass index (BMI) \|\| id:ukb-b-19953 | Coronary heart disease \|\| id:ieu-a-7 | Weighted median | 1.500729 | 2.99E-12 |
| Body mass index (BMI) \|\| id:ukb-b-19953 | Coronary heart disease \|\| id:ieu-a-7 | Weighted mode | 1.480569 | 0.000134 |
| Diastolic blood pressure automated reading \|\| id:ukb-a-359 | Coronary heart disease \|\| id:ieu-a-7 | Inverse variance weighted | 1.788093 | 3.32E-14 |
| Diastolic blood pressure automated reading \|\| id:ukb-a-359 | Coronary heart disease \|\| id:ieu-a-7 | MR Egger | 2.30741 | 0.003425 |
| Diastolic blood pressure automated reading \|\| id:ukb-a-359 | Coronary heart disease \|\| id:ieu-a-7 | Weighted median | 1.74836 | 1.27E-15 |
| Diastolic blood pressure automated reading \|\| id:ukb-a-359 | Coronary heart disease \|\| id:ieu-a-7 | Weighted mode | 1.721057 | 0.003377 |
| Systolic blood pressure automated reading \|\| id:ukb-a-360 | Coronary heart disease \|\| id:ieu-a-7 | Inverse variance weighted | 1.764253 | 4.35E-10 |
| Systolic blood pressure automated reading \|\| id:ukb-a-360 | Coronary heart disease \|\| id:ieu-a-7 | MR Egger | 2.634798 | 0.001195 |
| Systolic blood pressure automated reading \|\| id:ukb-a-360 | Coronary heart disease \|\| id:ieu-a-7 | Weighted median | 1.775096 | 6.79E-15 |
| Systolic blood pressure automated reading \|\| id:ukb-a-360 | Coronary heart disease \|\| id:ieu-a-7 | Weighted mode | 1.78212 | 0.001476 |
| Creatinine (enzymatic) in urine \|\| id:ukb-a-333 | Coronary heart disease \|\| id:ieu-a-7 | Inverse variance weighted | 1.398696 | 0.143798 |
| Creatinine (enzymatic) in urine \|\| id:ukb-a-333 | Coronary heart disease \|\| id:ieu-a-7 | MR Egger | 0.671919 | 0.710261 |
| Creatinine (enzymatic) in urine \|\| id:ukb-a-333 | Coronary heart disease \|\| id:ieu-a-7 | Weighted median | 1.774623 | 0.012535 |
| Creatinine (enzymatic) in urine \|\| id:ukb-a-333 | Coronary heart disease \|\| id:ieu-a-7 | Weighted mode | 1.95555 | 0.115262 |
| HDL cholesterol \|\| id:ieu-b-109 | Coronary heart disease \|\| id:ieu-a-7 | Inverse variance weighted | 0.773862 | 1.42E-11 |
| HDL cholesterol \|\| id:ieu-b-109 | Coronary heart disease \|\| id:ieu-a-7 | MR Egger | 0.86978 | 0.017567 |
| HDL cholesterol \|\| id:ieu-b-109 | Coronary heart disease \|\| id:ieu-a-7 | Weighted median | 0.800314 | 5.32E-07 |
| HDL cholesterol \|\| id:ieu-b-109 | Coronary heart disease \|\| id:ieu-a-7 | Weighted mode | 0.857742 | 0.007043 |
| LDL cholesterol \|\| id:ieu-b-110 | Coronary heart disease \|\| id:ieu-a-7 | Inverse variance weighted | 1.713871 | 1.05E-14 |
| LDL cholesterol \|\| id:ieu-b-110 | Coronary heart disease \|\| id:ieu-a-7 | MR Egger | 2.116592 | 9.38E-11 |
| LDL cholesterol \|\| id:ieu-b-110 | Coronary heart disease \|\| id:ieu-a-7 | Weighted median | 1.798876 | 7.34E-25 |
| LDL cholesterol \|\| id:ieu-b-110 | Coronary heart disease \|\| id:ieu-a-7 | Weighted mode | 1.965814 | 3.57E-19 |
| triglycerides \|\| id:ieu-b-111 | Coronary heart disease \|\| id:ieu-a-7 | Inverse variance weighted | 1.300392 | 3.77E-11 |
| triglycerides \|\| id:ieu-b-111 | Coronary heart disease \|\| id:ieu-a-7 | MR Egger | 1.172081 | 0.008181 |
| triglycerides \|\| id:ieu-b-111 | Coronary heart disease \|\| id:ieu-a-7 | Weighted median | 1.211477 | 0.000295 |
| triglycerides \|\| id:ieu-b-111 | Coronary heart disease \|\| id:ieu-a-7 | Weighted mode | 1.210286 | 7.75E-05 |
| Past tobacco smoking \|\| id:ukb-b-2134 | Coronary heart disease \|\| id:ieu-a-7 | Inverse variance weighted | 1.176193 | 0.037962 |
| Past tobacco smoking \|\| id:ukb-b-2134 | Coronary heart disease \|\| id:ieu-a-7 | MR Egger | 1.22401 | 0.611277 |
| Past tobacco smoking \|\| id:ukb-b-2134 | Coronary heart disease \|\| id:ieu-a-7 | Weighted median | 1.235918 | 0.016632 |
| Past tobacco smoking \|\| id:ukb-b-2134 | Coronary heart disease \|\| id:ieu-a-7 | Weighted mode | 1.430965 | 0.136858 |
| Sleep duration \|\| id:ukb-b-4424 | Coronary heart disease \|\| id:ieu-a-7 | Inverse variance weighted | 0.689252 | 0.009267 |
| Sleep duration \|\| id:ukb-b-4424 | Coronary heart disease \|\| id:ieu-a-7 | MR Egger | 0.748739 | 0.608138 |
| Sleep duration \|\| id:ukb-b-4424 | Coronary heart disease \|\| id:ieu-a-7 | Weighted median | 0.708072 | 0.063944 |
| Sleep duration \|\| id:ukb-b-4424 | Coronary heart disease \|\| id:ieu-a-7 | Weighted mode | 0.689503 | 0.250824 |
| Type 2 diabetes \|\| id:ieu-a-26 | Coronary heart disease \|\| id:ieu-a-7 | Inverse variance weighted | 1.101778 | 0.000158 |
| Type 2 diabetes \|\| id:ieu-a-26 | Coronary heart disease \|\| id:ieu-a-7 | MR Egger | 0.942542 | 0.684347 |
| Type 2 diabetes \|\| id:ieu-a-26 | Coronary heart disease \|\| id:ieu-a-7 | Weighted median | 1.10838 | 0.001645 |
| Type 2 diabetes \|\| id:ieu-a-26 | Coronary heart disease \|\| id:ieu-a-7 | Weighted mode | 1.105743 | 0.068515 |
| Number of days/week of vigorous physical activity 10+ minutes \|\| id:ukb-a-511 | Coronary heart disease \|\| id:ieu-a-7 | Inverse variance weighted | 1.020617 | 0.901838 |
| Number of days/week of vigorous physical activity 10+ minutes \|\| id:ukb-a-511 | Coronary heart disease \|\| id:ieu-a-7 | MR Egger | 0.011664 | 0.522345 |
| Number of days/week of vigorous physical activity 10+ minutes \|\| id:ukb-a-511 | Coronary heart disease \|\| id:ieu-a-7 | Weighted median | 1.082628 | 0.699278 |
| Number of days/week of vigorous physical activity 10+ minutes \|\| id:ukb-a-511 | Coronary heart disease \|\| id:ieu-a-7 | Weighted mode | 1.100662 | 0.738307 |
| Age when periods started (menarche) \|\| id:ukb-a-315 | Ischemic stroke (small-vessel) \|\| id:ebi-a-GCST006909 | Inverse variance weighted | 0.877177 | 0.309876 |
| Age when periods started (menarche) \|\| id:ukb-a-315 | Ischemic stroke (small-vessel) \|\| id:ebi-a-GCST006909 | MR Egger | 0.759852 | 0.469816 |
| Age when periods started (menarche) \|\| id:ukb-a-315 | Ischemic stroke (small-vessel) \|\| id:ebi-a-GCST006909 | Weighted median | 0.807697 | 0.256821 |
| Age when periods started (menarche) \|\| id:ukb-a-315 | Ischemic stroke (small-vessel) \|\| id:ebi-a-GCST006909 | Weighted mode | 0.802401 | 0.411817 |
| Alcohol consumption \|\| id:ieu-a-1283 | Ischemic stroke (small-vessel) \|\| id:ebi-a-GCST006909 | Inverse variance weighted | 0.844057 | 0.760244 |
| Alcohol consumption \|\| id:ieu-a-1283 | Ischemic stroke (small-vessel) \|\| id:ebi-a-GCST006909 | MR Egger | 0.153119 | 0.633859 |
| Alcohol consumption \|\| id:ieu-a-1283 | Ischemic stroke (small-vessel) \|\| id:ebi-a-GCST006909 | Weighted median | 0.911212 | 0.878879 |
| Alcohol consumption \|\| id:ieu-a-1283 | Ischemic stroke (small-vessel) \|\| id:ebi-a-GCST006909 | Weighted mode | 0.936642 | 0.927085 |
| Body mass index (BMI) \|\| id:ukb-b-19953 | Ischemic stroke (small-vessel) \|\| id:ebi-a-GCST006909 | Inverse variance weighted | 1.144308 | 0.08501 |
| Body mass index (BMI) \|\| id:ukb-b-19953 | Ischemic stroke (small-vessel) \|\| id:ebi-a-GCST006909 | MR Egger | 1.20987 | 0.375732 |
| Body mass index (BMI) \|\| id:ukb-b-19953 | Ischemic stroke (small-vessel) \|\| id:ebi-a-GCST006909 | Weighted median | 1.185533 | 0.219572 |
| Body mass index (BMI) \|\| id:ukb-b-19953 | Ischemic stroke (small-vessel) \|\| id:ebi-a-GCST006909 | Weighted mode | 1.186459 | 0.488975 |
| Diastolic blood pressure automated reading \|\| id:ukb-a-359 | Ischemic stroke (small-vessel) \|\| id:ebi-a-GCST006909 | Inverse variance weighted | 2.265269 | 5.73E-11 |
| Diastolic blood pressure automated reading \|\| id:ukb-a-359 | Ischemic stroke (small-vessel) \|\| id:ebi-a-GCST006909 | MR Egger | 2.023666 | 0.118106 |
| Diastolic blood pressure automated reading \|\| id:ukb-a-359 | Ischemic stroke (small-vessel) \|\| id:ebi-a-GCST006909 | Weighted median | 2.298456 | 1.71E-07 |
| Diastolic blood pressure automated reading \|\| id:ukb-a-359 | Ischemic stroke (small-vessel) \|\| id:ebi-a-GCST006909 | Weighted mode | 2.856517 | 0.011204 |
| Systolic blood pressure automated reading \|\| id:ukb-a-360 | Ischemic stroke (small-vessel) \|\| id:ebi-a-GCST006909 | Inverse variance weighted | 1.640271 | 0.000379 |
| Systolic blood pressure automated reading \|\| id:ukb-a-360 | Ischemic stroke (small-vessel) \|\| id:ebi-a-GCST006909 | MR Egger | 2.664668 | 0.035402 |
| Systolic blood pressure automated reading \|\| id:ukb-a-360 | Ischemic stroke (small-vessel) \|\| id:ebi-a-GCST006909 | Weighted median | 1.799627 | 0.000564 |
| Systolic blood pressure automated reading \|\| id:ukb-a-360 | Ischemic stroke (small-vessel) \|\| id:ebi-a-GCST006909 | Weighted mode | 1.868069 | 0.073782 |
| Creatinine (enzymatic) in urine \|\| id:ukb-a-333 | Ischemic stroke (small-vessel) \|\| id:ebi-a-GCST006909 | Inverse variance weighted | 1.530796 | 0.349021 |
| Creatinine (enzymatic) in urine \|\| id:ukb-a-333 | Ischemic stroke (small-vessel) \|\| id:ebi-a-GCST006909 | MR Egger | 3.944103 | 0.461411 |
| Creatinine (enzymatic) in urine \|\| id:ukb-a-333 | Ischemic stroke (small-vessel) \|\| id:ebi-a-GCST006909 | Weighted median | 2.304381 | 0.088567 |
| Creatinine (enzymatic) in urine \|\| id:ukb-a-333 | Ischemic stroke (small-vessel) \|\| id:ebi-a-GCST006909 | Weighted mode | 2.995395 | 0.113524 |
| HDL cholesterol \|\| id:ieu-b-109 | Ischemic stroke (small-vessel) \|\| id:ebi-a-GCST006909 | Inverse variance weighted | 0.844143 | 0.007753 |
| HDL cholesterol \|\| id:ieu-b-109 | Ischemic stroke (small-vessel) \|\| id:ebi-a-GCST006909 | MR Egger | 0.939407 | 0.52681 |
| HDL cholesterol \|\| id:ieu-b-109 | Ischemic stroke (small-vessel) \|\| id:ebi-a-GCST006909 | Weighted median | 0.93525 | 0.492779 |
| HDL cholesterol \|\| id:ieu-b-109 | Ischemic stroke (small-vessel) \|\| id:ebi-a-GCST006909 | Weighted mode | 0.962611 | 0.700404 |
| LDL cholesterol \|\| id:ieu-b-110 | Ischemic stroke (small-vessel) \|\| id:ebi-a-GCST006909 | Inverse variance weighted | 1.030177 | 0.726459 |
| LDL cholesterol \|\| id:ieu-b-110 | Ischemic stroke (small-vessel) \|\| id:ebi-a-GCST006909 | MR Egger | 1.153747 | 0.276356 |
| LDL cholesterol \|\| id:ieu-b-110 | Ischemic stroke (small-vessel) \|\| id:ebi-a-GCST006909 | Weighted median | 1.013955 | 0.902741 |
| LDL cholesterol \|\| id:ieu-b-110 | Ischemic stroke (small-vessel) \|\| id:ebi-a-GCST006909 | Weighted mode | 1.035501 | 0.746381 |
| triglycerides \|\| id:ieu-b-111 | Ischemic stroke (small-vessel) \|\| id:ebi-a-GCST006909 | Inverse variance weighted | 1.161273 | 0.025173 |
| triglycerides \|\| id:ieu-b-111 | Ischemic stroke (small-vessel) \|\| id:ebi-a-GCST006909 | MR Egger | 1.013361 | 0.895832 |
| triglycerides \|\| id:ieu-b-111 | Ischemic stroke (small-vessel) \|\| id:ebi-a-GCST006909 | Weighted median | 0.959675 | 0.675124 |
| triglycerides \|\| id:ieu-b-111 | Ischemic stroke (small-vessel) \|\| id:ebi-a-GCST006909 | Weighted mode | 0.971928 | 0.760332 |
| Past tobacco smoking \|\| id:ukb-b-2134 | Ischemic stroke (small-vessel) \|\| id:ebi-a-GCST006909 | Inverse variance weighted | 1.353212 | 0.038422 |
| Past tobacco smoking \|\| id:ukb-b-2134 | Ischemic stroke (small-vessel) \|\| id:ebi-a-GCST006909 | MR Egger | 3.832797 | 0.03617 |
| Past tobacco smoking \|\| id:ukb-b-2134 | Ischemic stroke (small-vessel) \|\| id:ebi-a-GCST006909 | Weighted median | 1.366818 | 0.131798 |
| Past tobacco smoking \|\| id:ukb-b-2134 | Ischemic stroke (small-vessel) \|\| id:ebi-a-GCST006909 | Weighted mode | 1.551984 | 0.331661 |
| Sleep duration \|\| id:ukb-b-4424 | Ischemic stroke (small-vessel) \|\| id:ebi-a-GCST006909 | Inverse variance weighted | 0.660208 | 0.16083 |
| Sleep duration \|\| id:ukb-b-4424 | Ischemic stroke (small-vessel) \|\| id:ebi-a-GCST006909 | MR Egger | 1.908104 | 0.574741 |
| Sleep duration \|\| id:ukb-b-4424 | Ischemic stroke (small-vessel) \|\| id:ebi-a-GCST006909 | Weighted median | 0.717565 | 0.422166 |
| Sleep duration \|\| id:ukb-b-4424 | Ischemic stroke (small-vessel) \|\| id:ebi-a-GCST006909 | Weighted mode | 0.853426 | 0.834467 |
| Type 2 diabetes \|\| id:ieu-a-26 | Ischemic stroke (small-vessel) \|\| id:ebi-a-GCST006909 | Inverse variance weighted | 1.085706 | 0.157453 |
| Type 2 diabetes \|\| id:ieu-a-26 | Ischemic stroke (small-vessel) \|\| id:ebi-a-GCST006909 | MR Egger | 0.976545 | 0.945431 |
| Type 2 diabetes \|\| id:ieu-a-26 | Ischemic stroke (small-vessel) \|\| id:ebi-a-GCST006909 | Weighted median | 1.128013 | 0.120357 |
| Type 2 diabetes \|\| id:ieu-a-26 | Ischemic stroke (small-vessel) \|\| id:ebi-a-GCST006909 | Weighted mode | 1.180669 | 0.183965 |
| Number of days/week of vigorous physical activity 10+ minutes \|\| id:ukb-a-511 | Ischemic stroke (small-vessel) \|\| id:ebi-a-GCST006909 | Inverse variance weighted | 1.347272 | 0.451056 |
| Number of days/week of vigorous physical activity 10+ minutes \|\| id:ukb-a-511 | Ischemic stroke (small-vessel) \|\| id:ebi-a-GCST006909 | MR Egger | 0.378111 | 0.948581 |
| Number of days/week of vigorous physical activity 10+ minutes \|\| id:ukb-a-511 | Ischemic stroke (small-vessel) \|\| id:ebi-a-GCST006909 | Weighted median | 1.196714 | 0.689201 |
| Number of days/week of vigorous physical activity 10+ minutes \|\| id:ukb-a-511 | Ischemic stroke (small-vessel) \|\| id:ebi-a-GCST006909 | Weighted mode | 0.874926 | 0.854134 |
| Age when periods started (menarche) \|\| id:ukb-a-315 | Ischemic stroke (large artery atherosclerosis) \|\| id:ebi-a-GCST006907 | Inverse variance weighted | 0.836082 | 0.19041 |
| Age when periods started (menarche) \|\| id:ukb-a-315 | Ischemic stroke (large artery atherosclerosis) \|\| id:ebi-a-GCST006907 | MR Egger | 0.984543 | 0.969259 |
| Age when periods started (menarche) \|\| id:ukb-a-315 | Ischemic stroke (large artery atherosclerosis) \|\| id:ebi-a-GCST006907 | Weighted median | 0.758668 | 0.18552 |
| Age when periods started (menarche) \|\| id:ukb-a-315 | Ischemic stroke (large artery atherosclerosis) \|\| id:ebi-a-GCST006907 | Weighted mode | 0.753186 | 0.371364 |
| Alcohol consumption \|\| id:ieu-a-1283 | Ischemic stroke (large artery atherosclerosis) \|\| id:ebi-a-GCST006907 | Inverse variance weighted | 2.448906 | 0.24151 |
| Alcohol consumption \|\| id:ieu-a-1283 | Ischemic stroke (large artery atherosclerosis) \|\| id:ebi-a-GCST006907 | MR Egger | 0.327542 | 0.855988 |
| Alcohol consumption \|\| id:ieu-a-1283 | Ischemic stroke (large artery atherosclerosis) \|\| id:ebi-a-GCST006907 | Weighted median | 4.154278 | 0.103435 |
| Alcohol consumption \|\| id:ieu-a-1283 | Ischemic stroke (large artery atherosclerosis) \|\| id:ebi-a-GCST006907 | Weighted mode | 8.209209 | 0.216449 |
| Body mass index (BMI) \|\| id:ukb-b-19953 | Ischemic stroke (large artery atherosclerosis) \|\| id:ebi-a-GCST006907 | Inverse variance weighted | 1.382207 | 0.000159 |
| Body mass index (BMI) \|\| id:ukb-b-19953 | Ischemic stroke (large artery atherosclerosis) \|\| id:ebi-a-GCST006907 | MR Egger | 1.434055 | 0.127165 |
| Body mass index (BMI) \|\| id:ukb-b-19953 | Ischemic stroke (large artery atherosclerosis) \|\| id:ebi-a-GCST006907 | Weighted median | 1.546273 | 0.00227 |
| Body mass index (BMI) \|\| id:ukb-b-19953 | Ischemic stroke (large artery atherosclerosis) \|\| id:ebi-a-GCST006907 | Weighted mode | 1.463007 | 0.124157 |
| Diastolic blood pressure automated reading \|\| id:ukb-a-359 | Ischemic stroke (large artery atherosclerosis) \|\| id:ebi-a-GCST006907 | Inverse variance weighted | 1.786436 | 1.12E-05 |
| Diastolic blood pressure automated reading \|\| id:ukb-a-359 | Ischemic stroke (large artery atherosclerosis) \|\| id:ebi-a-GCST006907 | MR Egger | 3.477916 | 0.009483 |
| Diastolic blood pressure automated reading \|\| id:ukb-a-359 | Ischemic stroke (large artery atherosclerosis) \|\| id:ebi-a-GCST006907 | Weighted median | 1.698461 | 0.002236 |
| Diastolic blood pressure automated reading \|\| id:ukb-a-359 | Ischemic stroke (large artery atherosclerosis) \|\| id:ebi-a-GCST006907 | Weighted mode | 1.996664 | 0.156775 |
| Systolic blood pressure automated reading \|\| id:ukb-a-360 | Ischemic stroke (large artery atherosclerosis) \|\| id:ebi-a-GCST006907 | Inverse variance weighted | 2.858081 | 9.07E-12 |
| Systolic blood pressure automated reading \|\| id:ukb-a-360 | Ischemic stroke (large artery atherosclerosis) \|\| id:ebi-a-GCST006907 | MR Egger | 5.207904 | 0.001474 |
| Systolic blood pressure automated reading \|\| id:ukb-a-360 | Ischemic stroke (large artery atherosclerosis) \|\| id:ebi-a-GCST006907 | Weighted median | 2.493044 | 6.47E-07 |
| Systolic blood pressure automated reading \|\| id:ukb-a-360 | Ischemic stroke (large artery atherosclerosis) \|\| id:ebi-a-GCST006907 | Weighted mode | 1.707215 | 0.286723 |
| Creatinine (enzymatic) in urine \|\| id:ukb-a-333 | Ischemic stroke (large artery atherosclerosis) \|\| id:ebi-a-GCST006907 | Inverse variance weighted | 0.913055 | 0.826008 |
| Creatinine (enzymatic) in urine \|\| id:ukb-a-333 | Ischemic stroke (large artery atherosclerosis) \|\| id:ebi-a-GCST006907 | MR Egger | 0.554167 | 0.732395 |
| Creatinine (enzymatic) in urine \|\| id:ukb-a-333 | Ischemic stroke (large artery atherosclerosis) \|\| id:ebi-a-GCST006907 | Weighted median | 0.587244 | 0.324848 |
| Creatinine (enzymatic) in urine \|\| id:ukb-a-333 | Ischemic stroke (large artery atherosclerosis) \|\| id:ebi-a-GCST006907 | Weighted mode | 0.408282 | 0.297346 |
| HDL cholesterol \|\| id:ieu-b-109 | Ischemic stroke (large artery atherosclerosis) \|\| id:ebi-a-GCST006907 | Inverse variance weighted | 0.83265 | 0.006619 |
| HDL cholesterol \|\| id:ieu-b-109 | Ischemic stroke (large artery atherosclerosis) \|\| id:ebi-a-GCST006907 | MR Egger | 1.050572 | 0.635375 |
| HDL cholesterol \|\| id:ieu-b-109 | Ischemic stroke (large artery atherosclerosis) \|\| id:ebi-a-GCST006907 | Weighted median | 1.059267 | 0.608052 |
| HDL cholesterol \|\| id:ieu-b-109 | Ischemic stroke (large artery atherosclerosis) \|\| id:ebi-a-GCST006907 | Weighted mode | 1.035637 | 0.724351 |
| LDL cholesterol \|\| id:ieu-b-110 | Ischemic stroke (large artery atherosclerosis) \|\| id:ebi-a-GCST006907 | Inverse variance weighted | 1.468815 | 9.66E-05 |
| LDL cholesterol \|\| id:ieu-b-110 | Ischemic stroke (large artery atherosclerosis) \|\| id:ebi-a-GCST006907 | MR Egger | 1.528383 | 0.006618 |
| LDL cholesterol \|\| id:ieu-b-110 | Ischemic stroke (large artery atherosclerosis) \|\| id:ebi-a-GCST006907 | Weighted median | 1.444811 | 0.006535 |
| LDL cholesterol \|\| id:ieu-b-110 | Ischemic stroke (large artery atherosclerosis) \|\| id:ebi-a-GCST006907 | Weighted mode | 1.315817 | 0.019392 |
| triglycerides \|\| id:ieu-b-111 | Ischemic stroke (large artery atherosclerosis) \|\| id:ebi-a-GCST006907 | Inverse variance weighted | 1.245659 | 0.001311 |
| triglycerides \|\| id:ieu-b-111 | Ischemic stroke (large artery atherosclerosis) \|\| id:ebi-a-GCST006907 | MR Egger | 1.079895 | 0.460562 |
| triglycerides \|\| id:ieu-b-111 | Ischemic stroke (large artery atherosclerosis) \|\| id:ebi-a-GCST006907 | Weighted median | 1.161358 | 0.166436 |
| triglycerides \|\| id:ieu-b-111 | Ischemic stroke (large artery atherosclerosis) \|\| id:ebi-a-GCST006907 | Weighted mode | 1.111577 | 0.326017 |
| Past tobacco smoking \|\| id:ukb-b-2134 | Ischemic stroke (large artery atherosclerosis) \|\| id:ebi-a-GCST006907 | Inverse variance weighted | 1.335631 | 0.077966 |
| Past tobacco smoking \|\| id:ukb-b-2134 | Ischemic stroke (large artery atherosclerosis) \|\| id:ebi-a-GCST006907 | MR Egger | 4.530056 | 0.03457 |
| Past tobacco smoking \|\| id:ukb-b-2134 | Ischemic stroke (large artery atherosclerosis) \|\| id:ebi-a-GCST006907 | Weighted median | 1.3217 | 0.209694 |
| Past tobacco smoking \|\| id:ukb-b-2134 | Ischemic stroke (large artery atherosclerosis) \|\| id:ebi-a-GCST006907 | Weighted mode | 1.42497 | 0.497941 |
| Sleep duration \|\| id:ukb-b-4424 | Ischemic stroke (large artery atherosclerosis) \|\| id:ebi-a-GCST006907 | Inverse variance weighted | 0.822251 | 0.620929 |
| Sleep duration \|\| id:ukb-b-4424 | Ischemic stroke (large artery atherosclerosis) \|\| id:ebi-a-GCST006907 | MR Egger | 0.675497 | 0.801111 |
| Sleep duration \|\| id:ukb-b-4424 | Ischemic stroke (large artery atherosclerosis) \|\| id:ebi-a-GCST006907 | Weighted median | 0.839063 | 0.707826 |
| Sleep duration \|\| id:ukb-b-4424 | Ischemic stroke (large artery atherosclerosis) \|\| id:ebi-a-GCST006907 | Weighted mode | 0.663846 | 0.592668 |
| Type 2 diabetes \|\| id:ieu-a-26 | Ischemic stroke (large artery atherosclerosis) \|\| id:ebi-a-GCST006907 | Inverse variance weighted | 1.081359 | 0.336614 |
| Type 2 diabetes \|\| id:ieu-a-26 | Ischemic stroke (large artery atherosclerosis) \|\| id:ebi-a-GCST006907 | MR Egger | 0.713397 | 0.478693 |
| Type 2 diabetes \|\| id:ieu-a-26 | Ischemic stroke (large artery atherosclerosis) \|\| id:ebi-a-GCST006907 | Weighted median | 1.02408 | 0.788579 |
| Type 2 diabetes \|\| id:ieu-a-26 | Ischemic stroke (large artery atherosclerosis) \|\| id:ebi-a-GCST006907 | Weighted mode | 1.0071 | 0.95076 |
| Number of days/week of vigorous physical activity 10+ minutes \|\| id:ukb-a-511 | Ischemic stroke (large artery atherosclerosis) \|\| id:ebi-a-GCST006907 | Inverse variance weighted | 0.924256 | 0.853271 |
| Number of days/week of vigorous physical activity 10+ minutes \|\| id:ukb-a-511 | Ischemic stroke (large artery atherosclerosis) \|\| id:ebi-a-GCST006907 | MR Egger | 59.43776 | 0.818411 |
| Number of days/week of vigorous physical activity 10+ minutes \|\| id:ukb-a-511 | Ischemic stroke (large artery atherosclerosis) \|\| id:ebi-a-GCST006907 | Weighted median | 0.962707 | 0.943202 |
| Number of days/week of vigorous physical activity 10+ minutes \|\| id:ukb-a-511 | Ischemic stroke (large artery atherosclerosis) \|\| id:ebi-a-GCST006907 | Weighted mode | 1.129547 | 0.884374 |
| Age when periods started (menarche) \|\| id:ukb-a-315 | Ischemic stroke (cardioembolic) \|\| id:ebi-a-GCST006910 | Inverse variance weighted | 1.250067 | 0.02844 |
| Age when periods started (menarche) \|\| id:ukb-a-315 | Ischemic stroke (cardioembolic) \|\| id:ebi-a-GCST006910 | MR Egger | 1.155949 | 0.631148 |
| Age when periods started (menarche) \|\| id:ukb-a-315 | Ischemic stroke (cardioembolic) \|\| id:ebi-a-GCST006910 | Weighted median | 1.213238 | 0.214113 |
| Age when periods started (menarche) \|\| id:ukb-a-315 | Ischemic stroke (cardioembolic) \|\| id:ebi-a-GCST006910 | Weighted mode | 1.215879 | 0.462001 |
| Alcohol consumption \|\| id:ieu-a-1283 | Ischemic stroke (cardioembolic) \|\| id:ebi-a-GCST006910 | Inverse variance weighted | 1.349776 | 0.528512 |
| Alcohol consumption \|\| id:ieu-a-1283 | Ischemic stroke (cardioembolic) \|\| id:ebi-a-GCST006910 | MR Egger | 17.04437 | 0.428259 |
| Alcohol consumption \|\| id:ieu-a-1283 | Ischemic stroke (cardioembolic) \|\| id:ebi-a-GCST006910 | Weighted median | 1.336329 | 0.592323 |
| Alcohol consumption \|\| id:ieu-a-1283 | Ischemic stroke (cardioembolic) \|\| id:ebi-a-GCST006910 | Weighted mode | 1.331315 | 0.640483 |
| Body mass index (BMI) \|\| id:ukb-b-19953 | Ischemic stroke (cardioembolic) \|\| id:ebi-a-GCST006910 | Inverse variance weighted | 1.14272 | 0.033724 |
| Body mass index (BMI) \|\| id:ukb-b-19953 | Ischemic stroke (cardioembolic) \|\| id:ebi-a-GCST006910 | MR Egger | 1.41841 | 0.043331 |
| Body mass index (BMI) \|\| id:ukb-b-19953 | Ischemic stroke (cardioembolic) \|\| id:ebi-a-GCST006910 | Weighted median | 1.230873 | 0.06833 |
| Body mass index (BMI) \|\| id:ukb-b-19953 | Ischemic stroke (cardioembolic) \|\| id:ebi-a-GCST006910 | Weighted mode | 1.239721 | 0.25135 |
| Diastolic blood pressure automated reading \|\| id:ukb-a-359 | Ischemic stroke (cardioembolic) \|\| id:ebi-a-GCST006910 | Inverse variance weighted | 1.392867 | 0.000276 |
| Diastolic blood pressure automated reading \|\| id:ukb-a-359 | Ischemic stroke (cardioembolic) \|\| id:ebi-a-GCST006910 | MR Egger | 1.546662 | 0.187378 |
| Diastolic blood pressure automated reading \|\| id:ukb-a-359 | Ischemic stroke (cardioembolic) \|\| id:ebi-a-GCST006910 | Weighted median | 1.558745 | 0.000512 |
| Diastolic blood pressure automated reading \|\| id:ukb-a-359 | Ischemic stroke (cardioembolic) \|\| id:ebi-a-GCST006910 | Weighted mode | 1.928145 | 0.122137 |
| Systolic blood pressure automated reading \|\| id:ukb-a-360 | Ischemic stroke (cardioembolic) \|\| id:ebi-a-GCST006910 | Inverse variance weighted | 1.297273 | 0.023571 |
| Systolic blood pressure automated reading \|\| id:ukb-a-360 | Ischemic stroke (cardioembolic) \|\| id:ebi-a-GCST006910 | MR Egger | 1.52337 | 0.275251 |
| Systolic blood pressure automated reading \|\| id:ukb-a-360 | Ischemic stroke (cardioembolic) \|\| id:ebi-a-GCST006910 | Weighted median | 1.384826 | 0.022612 |
| Systolic blood pressure automated reading \|\| id:ukb-a-360 | Ischemic stroke (cardioembolic) \|\| id:ebi-a-GCST006910 | Weighted mode | 1.572071 | 0.265414 |
| Creatinine (enzymatic) in urine \|\| id:ukb-a-333 | Ischemic stroke (cardioembolic) \|\| id:ebi-a-GCST006910 | Inverse variance weighted | 0.801364 | 0.442405 |
| Creatinine (enzymatic) in urine \|\| id:ukb-a-333 | Ischemic stroke (cardioembolic) \|\| id:ebi-a-GCST006910 | MR Egger | 1.110942 | 0.930255 |
| Creatinine (enzymatic) in urine \|\| id:ukb-a-333 | Ischemic stroke (cardioembolic) \|\| id:ebi-a-GCST006910 | Weighted median | 0.757023 | 0.50761 |
| Creatinine (enzymatic) in urine \|\| id:ukb-a-333 | Ischemic stroke (cardioembolic) \|\| id:ebi-a-GCST006910 | Weighted mode | 0.476488 | 0.388567 |
| HDL cholesterol \|\| id:ieu-b-109 | Ischemic stroke (cardioembolic) \|\| id:ebi-a-GCST006910 | Inverse variance weighted | 0.914368 | 0.061973 |
| HDL cholesterol \|\| id:ieu-b-109 | Ischemic stroke (cardioembolic) \|\| id:ebi-a-GCST006910 | MR Egger | 0.980722 | 0.795785 |
| HDL cholesterol \|\| id:ieu-b-109 | Ischemic stroke (cardioembolic) \|\| id:ebi-a-GCST006910 | Weighted median | 0.935319 | 0.454046 |
| HDL cholesterol \|\| id:ieu-b-109 | Ischemic stroke (cardioembolic) \|\| id:ebi-a-GCST006910 | Weighted mode | 0.941524 | 0.429111 |
| LDL cholesterol \|\| id:ieu-b-110 | Ischemic stroke (cardioembolic) \|\| id:ebi-a-GCST006910 | Inverse variance weighted | 1.039176 | 0.604361 |
| LDL cholesterol \|\| id:ieu-b-110 | Ischemic stroke (cardioembolic) \|\| id:ebi-a-GCST006910 | MR Egger | 1.245736 | 0.05768 |
| LDL cholesterol \|\| id:ieu-b-110 | Ischemic stroke (cardioembolic) \|\| id:ebi-a-GCST006910 | Weighted median | 1.079035 | 0.464603 |
| LDL cholesterol \|\| id:ieu-b-110 | Ischemic stroke (cardioembolic) \|\| id:ebi-a-GCST006910 | Weighted mode | 1.111929 | 0.259379 |
| triglycerides \|\| id:ieu-b-111 | Ischemic stroke (cardioembolic) \|\| id:ebi-a-GCST006910 | Inverse variance weighted | 0.957919 | 0.393681 |
| triglycerides \|\| id:ieu-b-111 | Ischemic stroke (cardioembolic) \|\| id:ebi-a-GCST006910 | MR Egger | 0.988398 | 0.880068 |
| triglycerides \|\| id:ieu-b-111 | Ischemic stroke (cardioembolic) \|\| id:ebi-a-GCST006910 | Weighted median | 0.910479 | 0.250393 |
| triglycerides \|\| id:ieu-b-111 | Ischemic stroke (cardioembolic) \|\| id:ebi-a-GCST006910 | Weighted mode | 0.935647 | 0.430428 |
| Past tobacco smoking \|\| id:ukb-b-2134 | Ischemic stroke (cardioembolic) \|\| id:ebi-a-GCST006910 | Inverse variance weighted | 1.09804 | 0.46563 |
| Past tobacco smoking \|\| id:ukb-b-2134 | Ischemic stroke (cardioembolic) \|\| id:ebi-a-GCST006910 | MR Egger | 1.841127 | 0.291172 |
| Past tobacco smoking \|\| id:ukb-b-2134 | Ischemic stroke (cardioembolic) \|\| id:ebi-a-GCST006910 | Weighted median | 1.272643 | 0.207809 |
| Past tobacco smoking \|\| id:ukb-b-2134 | Ischemic stroke (cardioembolic) \|\| id:ebi-a-GCST006910 | Weighted mode | 1.411017 | 0.322038 |
| Sleep duration \|\| id:ukb-b-4424 | Ischemic stroke (cardioembolic) \|\| id:ebi-a-GCST006910 | Inverse variance weighted | 0.947107 | 0.829618 |
| Sleep duration \|\| id:ukb-b-4424 | Ischemic stroke (cardioembolic) \|\| id:ebi-a-GCST006910 | MR Egger | 0.747816 | 0.770455 |
| Sleep duration \|\| id:ukb-b-4424 | Ischemic stroke (cardioembolic) \|\| id:ebi-a-GCST006910 | Weighted median | 0.868034 | 0.681279 |
| Sleep duration \|\| id:ukb-b-4424 | Ischemic stroke (cardioembolic) \|\| id:ebi-a-GCST006910 | Weighted mode | 0.881941 | 0.859052 |
| Type 2 diabetes \|\| id:ieu-a-26 | Ischemic stroke (cardioembolic) \|\| id:ebi-a-GCST006910 | Inverse variance weighted | 0.974051 | 0.695702 |
| Type 2 diabetes \|\| id:ieu-a-26 | Ischemic stroke (cardioembolic) \|\| id:ebi-a-GCST006910 | MR Egger | 0.637243 | 0.25463 |
| Type 2 diabetes \|\| id:ieu-a-26 | Ischemic stroke (cardioembolic) \|\| id:ebi-a-GCST006910 | Weighted median | 0.955804 | 0.536696 |
| Type 2 diabetes \|\| id:ieu-a-26 | Ischemic stroke (cardioembolic) \|\| id:ebi-a-GCST006910 | Weighted mode | 0.944026 | 0.575623 |
| Number of days/week of vigorous physical activity 10+ minutes \|\| id:ukb-a-511 | Ischemic stroke (cardioembolic) \|\| id:ebi-a-GCST006910 | Inverse variance weighted | 0.880366 | 0.709062 |
| Number of days/week of vigorous physical activity 10+ minutes \|\| id:ukb-a-511 | Ischemic stroke (cardioembolic) \|\| id:ebi-a-GCST006910 | MR Egger | 0.000826 | 0.648182 |
| Number of days/week of vigorous physical activity 10+ minutes \|\| id:ukb-a-511 | Ischemic stroke (cardioembolic) \|\| id:ebi-a-GCST006910 | Weighted median | 0.859949 | 0.711055 |
| Number of days/week of vigorous physical activity 10+ minutes \|\| id:ukb-a-511 | Ischemic stroke (cardioembolic) \|\| id:ebi-a-GCST006910 | Weighted mode | 0.812317 | 0.744011 |
